# Supplementary material for: Modeling glioblastoma heterogeneity as a dynamic network of cell states
Source: Mol Syst Biol. 2021 Sep 16;17(9):e10105. doi: 10.15252/msb.202010105 (PMC8444284; doi:10.15252/msb.202010105)
Supplement: Supplementary file 5 — Source Data for Figure 3 [file MSB-17-e10105-s001.zip › Figure3A_sourcedata/GSEA_3065/hallmarks_state3.GseaPreranked.1623416274744/HALLMARK_INTERFERON_GAMMA_RESPONSE.html]

Details for gene set HALLMARK\_INTERFERON\_GAMMA\_RESPONSE[GSEA]

|  || Dataset | state3 |
| Phenotype | NoPhenotypeAvailable |
| Upregulated in class | na\_neg |
| GeneSet | HALLMARK\_INTERFERON\_GAMMA\_RESPONSE |
| Enrichment Score (ES) | -0.24451542 |
| Normalized Enrichment Score (NES) | -0.8607022 |
| Nominal p-value | 0.74437296 |
| FDR q-value | 0.843846 |
| FWER p-Value | 1.0 |
Table: GSEA Results Summary

  

Fig 1: Enrichment plot: HALLMARK\_INTERFERON\_GAMMA\_RESPONSE      
 Profile of the Running ES Score & Positions of GeneSet Members on the Rank Ordered List

  

| PROBE | GENE SYMBOL | GENE\_TITLE | RANK IN GENE LIST | RANK METRIC SCORE | RUNNING ES | CORE ENRICHMENT || 1 | B2M |  |  | 11 | 0.538 | 0.0644 | No |
| 2 | TNFAIP6 |  |  | 158 | 0.307 | 0.0849 | No |
| 3 | LGALS3BP |  |  | 439 | 0.211 | 0.0784 | No |
| 4 | CD74 |  |  | 471 | 0.204 | 0.0996 | No |
| 5 | PARP14 |  |  | 664 | 0.176 | 0.0990 | No |
| 6 | SPPL2A |  |  | 870 | 0.151 | 0.0937 | No |
| 7 | RNF213 |  |  | 964 | 0.141 | 0.1002 | No |
| 8 | STAT3 |  |  | 965 | 0.141 | 0.1174 | No |
| 9 | ADAR |  |  | 991 | 0.139 | 0.1314 | No |
| 10 | PNPT1 |  |  | 1046 | 0.134 | 0.1416 | No |
| 11 | TAPBP |  |  | 1055 | 0.134 | 0.1570 | No |
| 12 | TXNIP |  |  | 1317 | 0.114 | 0.1407 | No |
| 13 | TOR1B |  |  | 1700 | 0.092 | 0.1078 | No |
| 14 | HELZ2 |  |  | 1780 | 0.087 | 0.1093 | No |
| 15 | ARID5B |  |  | 1833 | 0.085 | 0.1137 | No |
| 16 | ST3GAL5 |  |  | 2139 | 0.071 | 0.0870 | No |
| 17 | IFNAR2 |  |  | 2161 | 0.070 | 0.0931 | No |
| 18 | TAP1 |  |  | 2349 | 0.063 | 0.0792 | No |
| 19 | PTPN2 |  |  | 2365 | 0.062 | 0.0851 | No |
| 20 | OAS3 |  |  | 2397 | 0.061 | 0.0889 | No |
| 21 | C1R |  |  | 2407 | 0.060 | 0.0952 | No |
| 22 | BST2 |  |  | 2410 | 0.060 | 0.1024 | No |
| 23 | SAMD9L |  |  | 2442 | 0.059 | 0.1060 | No |
| 24 | PDE4B |  |  | 2443 | 0.059 | 0.1132 | No |
| 25 | NAMPT |  |  | 2583 | 0.054 | 0.1037 | No |
| 26 | DDX60 |  |  | 2612 | 0.053 | 0.1070 | No |
| 27 | HIF1A |  |  | 2661 | 0.051 | 0.1077 | No |
| 28 | STAT2 |  |  | 2749 | 0.049 | 0.1036 | No |
| 29 | PSMA2 |  |  | 3044 | 0.040 | 0.0745 | No |
| 30 | TRIM25 |  |  | 3298 | 0.033 | 0.0493 | No |
| 31 | NUP93 |  |  | 3370 | 0.031 | 0.0450 | No |
| 32 | IFITM3 |  |  | 3449 | 0.029 | 0.0395 | No |
| 33 | IRF1 |  |  | 3457 | 0.029 | 0.0423 | No |
| 34 | IRF7 |  |  | 3530 | 0.027 | 0.0373 | No |
| 35 | AUTS2 |  |  | 3548 | 0.027 | 0.0386 | No |
| 36 | CMTR1 |  |  | 3876 | 0.018 | 0.0030 | No |
| 37 | OGFR |  |  | 3904 | 0.017 | 0.0020 | No |
| 38 | TRIM26 |  |  | 3984 | 0.016 | -0.0052 | No |
| 39 | PFKP |  |  | 4040 | 0.014 | -0.0098 | No |
| 40 | NCOA3 |  |  | 4125 | 0.012 | -0.0181 | No |
| 41 | PML |  |  | 4353 | 0.005 | -0.0436 | No |
| 42 | PLSCR1 |  |  | 4373 | 0.005 | -0.0453 | No |
| 43 | SAMHD1 |  |  | 4378 | 0.004 | -0.0452 | No |
| 44 | SSPN |  |  | 4525 | 0.001 | -0.0620 | No |
| 45 | NFKB1 |  |  | 4528 | 0.001 | -0.0621 | No |
| 46 | LAP3 |  |  | 4547 | -0.000 | -0.0642 | No |
| 47 | STAT1 |  |  | 4591 | -0.001 | -0.0690 | No |
| 48 | LY6E |  |  | 4707 | -0.004 | -0.0819 | No |
| 49 | TRAFD1 |  |  | 4779 | -0.006 | -0.0894 | No |
| 50 | CASP4 |  |  | 4831 | -0.007 | -0.0944 | No |
| 51 | RIPK1 |  |  | 5070 | -0.013 | -0.1204 | No |
| 52 | TRIM14 |  |  | 5086 | -0.013 | -0.1205 | No |
| 53 | IFITM2 |  |  | 5106 | -0.014 | -0.1210 | No |
| 54 | IFI44 |  |  | 5233 | -0.017 | -0.1336 | No |
| 55 | SLC25A28 |  |  | 5337 | -0.019 | -0.1431 | No |
| 56 | SOCS3 |  |  | 5338 | -0.019 | -0.1407 | No |
| 57 | LYSMD2 |  |  | 5367 | -0.020 | -0.1415 | No |
| 58 | ZNFX1 |  |  | 5414 | -0.021 | -0.1442 | No |
| 59 | PSMA3 |  |  | 5498 | -0.024 | -0.1509 | No |
| 60 | SP110 |  |  | 5521 | -0.025 | -0.1504 | No |
| 61 | NMI |  |  | 5592 | -0.027 | -0.1552 | No |
| 62 | MVP |  |  | 5610 | -0.028 | -0.1538 | No |
| 63 | NFKBIA |  |  | 5684 | -0.029 | -0.1586 | No |
| 64 | APOL6 |  |  | 5776 | -0.032 | -0.1652 | No |
| 65 | SOD2 |  |  | 5817 | -0.033 | -0.1658 | No |
| 66 | PTPN1 |  |  | 6055 | -0.041 | -0.1881 | No |
| 67 | EIF2AK2 |  |  | 6220 | -0.047 | -0.2013 | No |
| 68 | PSMB8 |  |  | 6287 | -0.049 | -0.2029 | No |
| 69 | ISG20 |  |  | 6399 | -0.053 | -0.2093 | No |
| 70 | ISOC1 |  |  | 6447 | -0.054 | -0.2081 | No |
| 71 | USP18 |  |  | 6464 | -0.055 | -0.2033 | No |
| 72 | METTL7B |  |  | 6482 | -0.056 | -0.1984 | No |
| 73 | CASP7 |  |  | 6580 | -0.060 | -0.2023 | No |
| 74 | IRF2 |  |  | 6602 | -0.061 | -0.1973 | No |
| 75 | RIPK2 |  |  | 6615 | -0.062 | -0.1912 | No |
| 76 | MYD88 |  |  | 6658 | -0.064 | -0.1882 | No |
| 77 | MTHFD2 |  |  | 6745 | -0.068 | -0.1899 | No |
| 78 | PSMB2 |  |  | 6885 | -0.075 | -0.1967 | No |
| 79 | CASP3 |  |  | 6969 | -0.079 | -0.1967 | No |
| 80 | IFI35 |  |  | 7384 | -0.107 | -0.2315 | Yes |
| 81 | PSME2 |  |  | 7389 | -0.107 | -0.2188 | Yes |
| 82 | PSME1 |  |  | 7399 | -0.108 | -0.2067 | Yes |
| 83 | UBE2L6 |  |  | 7684 | -0.137 | -0.2227 | Yes |
| 84 | ARL4A |  |  | 7829 | -0.155 | -0.2204 | Yes |
| 85 | PSMB9 |  |  | 7835 | -0.156 | -0.2019 | Yes |
| 86 | RBCK1 |  |  | 7907 | -0.167 | -0.1897 | Yes |
| 87 | BPGM |  |  | 8071 | -0.197 | -0.1845 | Yes |
| 88 | ISG15 |  |  | 8104 | -0.205 | -0.1632 | Yes |
| 89 | BTG1 |  |  | 8162 | -0.218 | -0.1431 | Yes |
| 90 | UPP1 |  |  | 8293 | -0.256 | -0.1268 | Yes |
| 91 | VAMP5 |  |  | 8312 | -0.260 | -0.0971 | Yes |
| 92 | SRI |  |  | 8395 | -0.292 | -0.0709 | Yes |
| 93 | MT2A |  |  | 8650 | -0.456 | -0.0447 | Yes |
| 94 | CDKN1A |  |  | 8657 | -0.461 | 0.0109 | Yes |
Table: GSEA details [plain text format]

  

Fig 2: HALLMARK\_INTERFERON\_GAMMA\_RESPONSE: Random ES distribution      
 Gene set null distribution of ES for **HALLMARK\_INTERFERON\_GAMMA\_RESPONSE**
